# Surgical approaches for treatment of ureteropelvic junction obstruction – a systematic review and network meta-analysis

**DOI:** 10.1186/s12894-019-0544-7

**Published:** 2019-11-11

**Authors:** Annemarie Uhlig, Johannes Uhlig, Lutz Trojan, Marc Hinterthaner, Alexander von Hammerstein-Equord, Arne Strauss

**Affiliations:** 1 Department of Urology, University Medical Center Goettingen, Goettingen, Germany; 2 Department of Diagnostic and Interventional Radiology, University Medical Center Goettingen, Goettingen, Germany; 3 Division of Interventional Radiology, Department of Radiology and Biomedical Imaging, Yale School of Medicine, New Haven, CT USA; 4 Department of Thoracic and Cardiovascular Surgery, University Medical Center Goettingen, Goettingen, Germany

**Keywords:** Endoscopy, Laparoscopy, Minimally invasive surgical procedures, Laparotomy, Network meta-analysis

## Abstract

**Background:**

Multiple surgical treatment options are available for the treatment of ureteropelvic junction obstruction (UPJO). The aim of this study is to compare the most frequently used technics in a comprehensive network approach.

**Methods:**

A systematic literature search of the EMBASE, MEDLINE and COCHRANE libraries was conducted in January 2018. Publications were included that evaluated at least two of the following surgical techniques: open pyeloplasty (OP), endopyelotomy (EP), laparoscopic (LP) and robot assisted pyeloplasty (RP). Main outcomes were operative success, complications, urinary leakage, re-operation, transfusion rate, operating time, and length of stay. Network meta-analyses with random effects models simultaneously assessed effectiveness of all surgical techniques.

**Results:**

A total of 26 studies including 3143 patients were analyzed. Compared with RP, EP and LP showed lower operative success rates (EP: OR = 0.09, 95%CI:0.05–0.19; *p* < 0.001; LP: OR = 0.51, 95%CI:0.31–0.84; *p* = 0.008). Compared with OP, LP and RP had lower risk for complications (LP: OR = 0.62; 95%CI:0.41–0.95; *p* = 0.027; RP: OR = 0.41; 95%CI:0.22–0.79; *p* = 0.007). Compared with RP, no significant differences were detected for urinary leakage or re-operation, transfusion rates. Compared with EP, RP yielded longer operating time (mean = 102.87 min, 95%CI:41.79 min–163.95 min, *p* = < 0.001). Further significant differences in operating times were detected when comparing LP to EP (mean = 115.13 min, 95%CI:65.63 min–164.63 min, *p* = < 0.001) and OP to EP (mean = 91.96 min, 95%CI:32.33 min–151.58 min, *p* = 0.003).

**Conclusions:**

Multiple surgical techniques are available for treatment of UPJO. RP has the highest rates of operative success and as well as LP lower complication rates than OP. Although surgical outcomes are worse for EP, its operating time is shorter than OP, RP, and LP. Surgeons should consider these findings when selecting the optimal treatment method for individual patients.

## Background

Surgical techniques for treatment of ureteropelvic junction obstruction (UPJO) have seen significant advancements during the last decades. From open approaches over laparoscopic techniques and endopyelotomy (EP) in the 1990s, robot assisted approaches have been introduced in 2002 [1, 2]. Currently, all of these techniques are clinically applied, although laparoscopic and robot assisted pyeloplasty (LP and RP) are often claimed to be superior to other approaches [3–7]. Several studies reported that minimal invasive treatment options outperform open pyeloplasty (OP) with respect to early recovery and lower complication rates, whereas OP operating time is shorter [3, 4]. Comparing LP and RP, length of hospital stay, operating and suturing time seem to be shorter for RP [8–11]. In one meta-analysis, operative success rate was significantly higher for RP [9]. While abdominal surgery is required for open, laparoscopic and robot assisted approaches, EP probably has the lowest invasiveness [12]. Even though EP has been replaced by other approaches in many institutions, several experts in the field still advocate this technique [13]; especially, as few literature is available summarizing the evidence on all approaches. Notwithstanding that meta-analyses have been published comparing individual studies for two treatment options, these provides limited guidance in the current situation with multiple approaches available for UPJO which must be evaluated against each another. The aim of this network-meat analysis is to provide a comprehensive overview for most frequently used techniques for treatment of UPJO and to compare their effectiveness regarding various clinically important outcomes.

## Materials and methods

### Search strategy

This study was registered a priori at PROSPERO (CRD42018085917). The systematic literature search using EMBASE, MEDLINE and COCHRANE libraries was performed in January 2018 and was unrestricted with respect to publication language and date. In addition, publication lists of reviews and included articles as well as conference proceedings were searched. Furthermore, we searched a registry for clinical trials (www.clinicaltrials.gov) to identify potentially unpublished studies. The full search strategy is available in the Additional file 1.

### Criteria for study inclusion and exclusion

We included retro- and prospective studies comparing at least two of the following surgical approaches for the treatment of UPJO: OP, LP, RP and EP. Studies had to assess at least one of the following outcomes: operative success, operating time, suturing time, estimated blood loss, transfusion rate, conversion rate, re-operation rate, intra- and postoperative as well as overall complications, urinary leakage, deaths, length of hospital stay, time to return to normal activities, renal function, pain, analgesia requirement, survival time and costs. No minimum follow-up time was required for study inclusion. Of these, the following outcomes were statistically evaluated: operative success was examined as the total number of successes as defined by the authors; rates of transfusion, conversion, re-operation as well as intra- and postoperative complications, and urinary leakage were evaluated as total numbers as well. Reviews and meta-analyses were excluded as well as studies focusing on children or animals. In addition, we excluded studies which reported insufficient data on measures of dispersion or pooled outcome data of two surgical approaches. In case of more than one publication reporting on the same patient cohort, the more comprehensive one was selected in order to meet the assumption of independence for meta-analyses.

### Definition of operative success

The authors of the papers used varying definitions for operative success: Objective success was reported by all authors and included mostly included patent ureteropelvic junction confirmed by radionuclide diuretic renogram or intravenous urography (IVU) and sometimes decrease in severity of hydronephrosis. Objective success was often defined as absence of symptoms or “significant” improvement with no further specification. If separate measures for subjective and objective success were reported instead of combined values, only success rates of objective measures were included in the in the statistical analysis to account for the subjectivity of perceived pain [14–16]. Measures for success of the surgical procedure e.g. whether a laparoscopic surgery could be performed without conversion, were not taken into account.

### Data extraction

Publication titles identified via the literature search were independently screened by 3 blinded authors resulting in a selection for abstract and full text screening which was performed by 2 independent blinded authors. From the resulting list of publications suited for inclusion, data extraction was performed in the same manner. Disagreement was resolved by consultation of a third author and majority vote. As suggested by Rothman et al., a study was considered as prospective if data collection on interventions and covariates took place before the outcome occurred [17].

### Assessment of study quality

Study quality assessment using the Downs and Black instrument was performed by two independent blinded authors resolving disagreement by consensus involving a third author [18]. The Downs and Black instrument rates the quality of randomized and non-randomized studies on a scale from 0 to 32 points (0 points for the worst and 32 for the best study quality) using a catalogue of 27 items. For each item 1 point is given except for the description of the distribution of principal confounders in each group of subjects where a maximum of 2 points can be reached and for the evaluation of study power for which a maximum of 5 points can be reached. For power evaluation study sizes were credited 1 up to 5 points for < 15, 15 to 44, 45 to 59, 60 to 100, > 100 patients according to the quartiles of sample sizes of included studies. Study quality was labeled “low” (1–10 points on Downs and Black instrument), “moderate” (11–21 points) and “high” (22–32 points). The Newcastle Ottawa Scale was used for additional study quality assessment. As suggested by the Cochrane handbook, study quality was separately assessed for each outcome [19].

### Statistical analyses

For dichotomous outcomes, Odds Ratios (OR) were calculated from absolute numbers or percentage given in standard manner. Continuous outcomes such as operating time, estimated blood loss and length of hospital stay were compared as median or mean with standard deviation. If available, data from multivariable models was preferentially used [20, 21]. In case of two treatment for similar interventions e.g. endopyelotomy and acucise endopyelotomy, the results were pooled [22]. If only median and interquartile range (IQR) for continuous outcomes were reported, and a large sample size indicated the high probability of an underlying normal distribution, the standard deviation was calculated by dividing the IQR by 1.35 [16]. All outcomes were compared to endopyelotomy as the reference group. A network meta-analysis with random effects approach was used as statistical method for comparison. All outcomes were ranked by p-score methods to estimate the amount of certainty that a single treatment outperforms the average of competing interventions. The p-score ranges from 0 to 1, the latter indicating the highest certainty possible [23]. Study heterogeneity was evaluated by Higgins’s I^2^ considering percentages below 25% as of potentially low relevance, from 26 to 50% as “moderate”, from 51 to 75% as “substantial”, and from 76 to 100% as “considerable” heterogeneity [19]. The consistency assumption was evaluated via visual assessment of net heat plots and by Cochran’s Q statistic. All pairwise comparisons of more than 10 studies were tested for publication bias using the weighted linear regression of the treatment effect on its standard error [24]. Sensitivity analyses were performed including only studies with at least 12 months follow-up time. The statistical analysis was performed using R version 3.4.2 with the packages “meta”, “netmeta”, and “metabias” as well as R Studio version 1.1.383. All *p*-values were calculated two-sided and an alpha-level of < 0.05 was considered statistically significant.

## Results

### Study characteristics

**Fig. 1:**
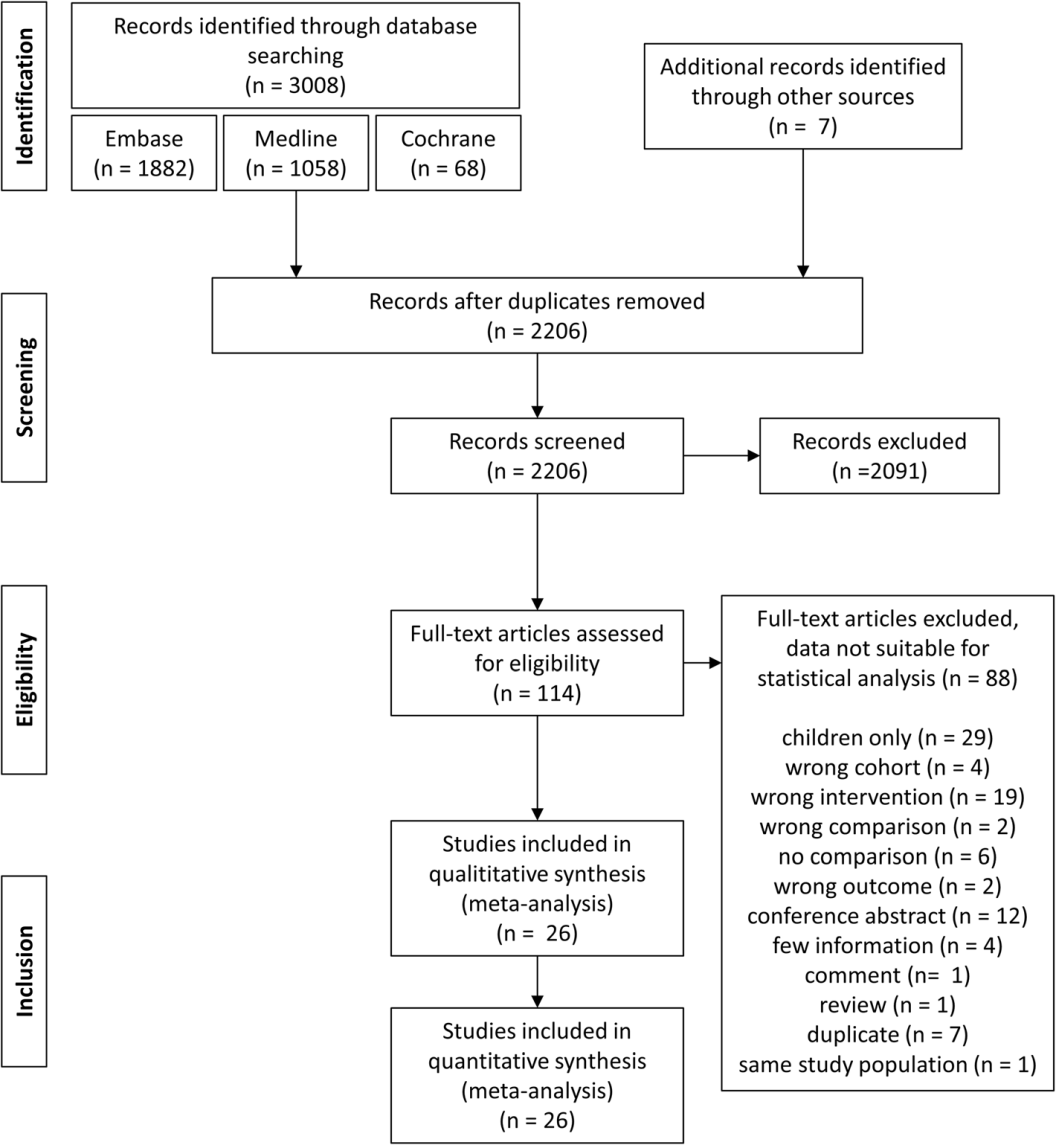
Flow diagram of study inclusion process

**Fig. 2:**
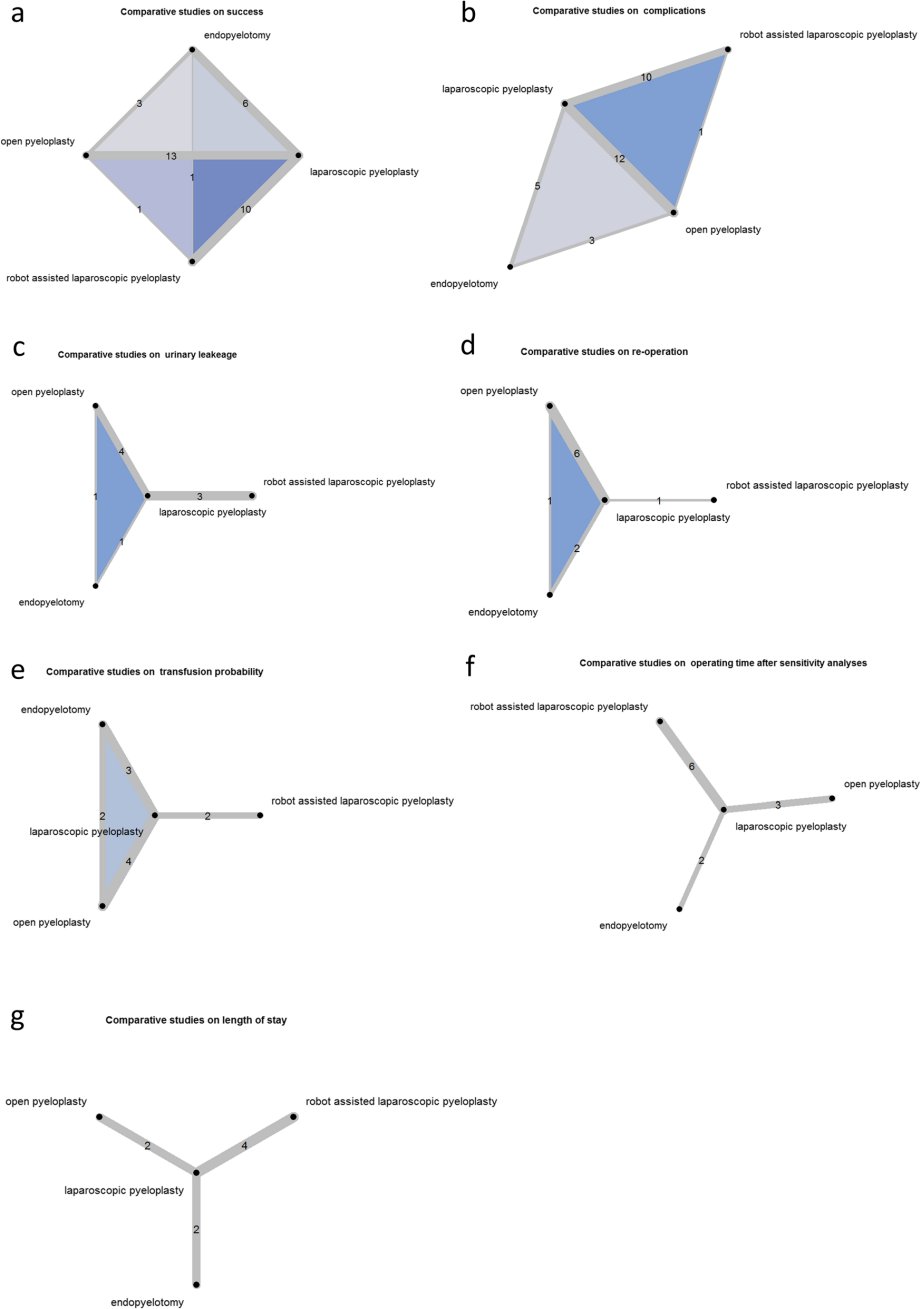
Number of comparisons for each end point. **a**) Comparative studies on success. **b**) Comparative studies on complications. **c**) Comparative studies on urinary leakeage. **d**) Comparative studies on re-operation. **e**) Comparative studies on transfusion probability. **f**) Comparative studies on operating time after sensitivity analyses. **g**) Comparative studies on length of stay

The systematic literature search yielded 3008 studies published between 1995 and 2017 of which 26 fulfilled the inclusion criteria [14–16, 20–22, 25–44]. Figure 1 depicts the selection process of the studies whereas Table 1 details the study characteristics. In total, 3143 patients were analyzed: 556 receiving OP, 1540 receiving LP, 798 receiving RP, and 249 receiving EP. Operative success and complications were evaluated by 24 studies each, whereas operating time was reported by 22 studies and length of hospital stay by 21 studies. Estimated blood loss was evaluated by 13 studies, postoperative complications by 10 studies, conversion rates by 9 studies and re-operation rates by 8 studies. Transfusion rates, intraoperative complications, urinary leakage and analgesia requirement were reported by 7 studies each. Four studies evaluated death rates, whereas suturing time and time to return to normal activities was reported by 3 studies each. Pain, renal function, and costs were evaluated by 1 study each whilst none of the included studies reported on survival time. Six studies were three-armed trials [14, 21, 25, 28, 30, 40]; the remaining 20 studies evaluated two of the 4 outcomes. Figure 2 details the number of comparisons for each end point. Only 3 congress abstracts provided sufficient data to meet the inclusion criteria [30, 31, 38]. Study design was retrospective in 18 studies, 1 study included pre- and retrospective data [40] and 5 studies explicitly stated a prospective design [27, 36, 38, 41, 44]. For 2 studies no information was available [31, 32]. Only one study had a randomized controlled design [38] and two further studies used multivariable adjustment methods to account for confounding [20, 21]. The mean follow up ranged from 1 month to greater than 6 years with the majority of the studies reporting follow-up times greater than 1 year. The study population consisted of adult patients in 15 of the included publications, whereas 8 studies reported on mixed cohorts of adults and children with the majority of patients being of age > 18 years in case of available information [30, 32–34, 41–44]. For 3 studies the inclusion criteria with respect to patient age were unclear [21, 31, 38]. The percentage of female patients ranged from 32 to 77% with 6 studies omitting this information [15, 16, 25, 26, 31, 38]. The geographic region of the study population was Asia in 8 cases [22, 30, 32, 34, 37, 40, 41, 43], Europe for 7 studies [15, 29, 31, 33, 35, 38, 42], and North America for 10 studies [16, 20, 21, 25–28, 36, 39, 44]. One study reported on a mixed Asian and North American population [14].

**Table 1:** Study characteristics of included studies

Author	Publication year	Design	Operative approaches	End points	Study size	Secondary UPJO	Uro-lithiasis	Country	Population	Mean age [years]	Percent female	Follow up [monts]
Baldwin et al. [25]	2003	retrospective	EP, LP, OP	operative success, operating time, estimated blood loss, transfusion, complications, length of stay, analgesia requirement	32	LP 44%, EP 33%, OP 43%	NA	USA	adult	44,4 and 37 and 42	NA	11,0 and 9,9 and 5,4
Bernie et al. [26]	2005	retrospective	LP, RP	operative success, operating time, estimated blood loss, complications, urinary leakage, length of stay	14	NA	NA	USA	adult	34 and 32	NA	24 and 10
Bird et al. [27]	2011	prospective	LP, RP	operative success, operating time, suturing time, estimated blood loss, conversion, complications, length of stay	172	13% overall	LP 16%, RP 30%	USA	adult	39,7+/−14,6	53	NA
Brooks et al. [28]	1995	retrospective	EP, LP, OP	operative success, operating time, transfusion, re-operation, complications, length of stay, time to return to normal activity, analgesia requirement	45	EP 23%, LP 8%, OP 18%	EP 1%, LP 0%, OP 9%	USA	adult	42 and 38,4 and 30,6	60	22,0 and 13,6 and 26,0
Calvert et al. [29]	2008	retrospective	LP, OP	operative success, operating time, conversion, complications, length of stay	100	OP 6%, LP 14%	NA	UK	adult	36,5	77	9 and 12
Chen et al. [30]	2016	retrospective	EP, LP, OP	operative success, operating time, estimated blood loss, conversion, complications, urinary leakage, length of stay	109	NA	NA	China	children and adult	32,8+/−15,6 and 30,9+/− 12,9 and 37,5+/− 12,0	39	51,9+/− 40,1
Danuser et al. [31]	2012	NA	LP, RP	operative success, operating time, conversion, complications, length of stay	82	NA	NA	Switzerland	NA	NA	NA	NA
Desai et al. [14]	2004	retrospective	EP, LP	operative success, operating time, re-operation, complications, length of stay	29	none	NA	USA and India	adult	38,6+/−16,1 and 38,9+/− 17	48	31,4+/− 12,6 and 20,0+/−14,0
Fahad [32]	2017	NA	LP, OP	operative success, operating time, conversion, re-operation, complications, urinary leakage, deaths, length of stay, time to return to normal activity, analgesia requirement	40	NA	NA	Iraq	children and adult	24	35	9
Garcia-Galisteo et al. [33]	2011	retrospective	LP, RP	operative success, operating time, suturing time, estimated blood loss, transfusion, complications, deaths, length of stay	50	NA	NA	Spain	children and adult	33,9	60	20,6 and 42,5
Han et al. [22]	2008	retrospective	EP, LP	operative success, operating time, transfusion, complications, length of stay, renal function	45	EP 31%, LP 13%	NA	Korea	adult	45,22 and 49,5 ± 13,6	47	15,2 and 14,3
Hanske et al. [20]	2015	retrospective	LP, OP	transfusion, re-operation, complications, deaths	593	NA	NA	USA	adult	median 48	57	1
Hemal et al. [34]	2010	retrospective	LP, RP	operative success, operating time, suturing time, estimated blood loss, conversion, re-operation, complications, length of stay, time to return to normal activity, analgesia requirement	60	NA	NA	India	children and adult	24,9 and 28,1	32	18
Klingler et al. [35]	2003	retrospective	LP, OP	operative success, re-operation, complications, pain, analgesia requirement	55	LP 8%, OP 0%	NA	Switzerland	adult	35,9+/−21,1 and 41,0+/−27,9	60	19,4+/−9,1 and 17,9+/−8,8
Link et al. [36]	2006	prospective	LP, RP	operating time, complications, urinary leakage	20	none	NA	USA	adult	46,5 and 38,0	65	5,6
Lucas et al. [16]	2012	retrospective	LP, RP	operative success, operating time, estimated blood loss, complications, urinary leakage	759	RP 27%, LP 24%	RP 18%, LP 12%	North America	NA	35	NA	11+/−13 and 15+/− 16
Memon et al. [37]	2016	retrospective	LP, OP	operative success, operating time, estimated blood loss, complications, length of stay	73	none	NA	Pakistan	adult	28 and 30	59	2,7+/−1,2
Mohammed [38]	2017	prospective	LP, OP	operative success, operating time, estimated blood loss, re-operation, length of stay	55	NA	NA	Germany	obese, NA	NA	NA	> 12
Olweny et al. [39]	2012	retrospective	LP, RP	operative success, operating time, estimated blood loss, conversion, complications, length of stay, analgesia requirement	20	none	NA	USA	adult	40,3 and 35,8	57	2,8 and 9,2
Pahwa et al. [40]	2014	pro- and retrospective	LP, OP, RP	operative success, operating time, estimated blood loss, complications, length of stay	90	none	NA	India	adult	31,4 and 34,4 and 32	33	32,0 and 18,0 and 13,5
Rivas et al. [15]	2015	retrospective	LP, OP	operative success, conversion, complications, deaths, length of stay	92	NA	LP 19%, OP 20%	Spain	adult	44,5 and 38,5	NA	45
Simforoosh et al. [41]	2004	prospective	LP, OP	operative success, operating time, transfusion, re-operation, complications, urinary leakage, length of stay	69	none	NA	Iran	children and adult	18,2 and 23,1	38	16,5 and 11,4
Umari et al. [42]	2011	retrospective	LP, OP	operative success, operating time, conversion, complications, length of stay	49	NA	LP 17%, OP 32%	Italy	children and adult	42	51	72,3 and 40,9
Wang et al. [43]	2013	retrospective	LP, OP	operative success, operating time, estimated blood loss, complications, urinary leakage, length of stay, analgesia requirement	172	NA	NA	China	children and adult	21 AND 25 years	34	32,7 AND 38,4 months
Weise and Winfield [44]	2006	prospective	LP, RP	operative success, operating time, estimated blood loss, transfusion, complications, length of stay	45	LP 7%, RP 0%	NA	USA	children and adult	26 and 24,5	59	6 and 10
Yanke et al. [21]	2008	retrospective	EP, LP, RP	operative success	273	EP 29%; LP 28%, RP 31%	NA	USA	adult	NA	61	20 and 20 and 19

### Study quality

The median study quality was 14 points (range from 6 to 25 points). Reasons for the moderate quality were missing randomization, allocation concealment and blinding as well insufficient confounder adjustment and loss to follow-up in almost all studies. Nevertheless, many studies clearly described study hypothesis, main outcomes and findings as well as patient characteristics and selected participants representative for the source population. Table 2 details the study quality separately for the most important endpoints success and complications. Comparable results were obtained using the Newcastle Ottawa scale (Additional file 1: Table S3).

**Table 2:** study quality according to the Downs and Black instrument for success and complications

	Baldwin et al. [25]	Bernie et al. [26]	Bird et al. [27]	Brooks et al. [28]	Calvert et al. [29]	Chen et al. [30]	Danuser et al. [31]	Desai et al. [14]	Fahad [32]	Garcia-Galisteo et al. [33]	Han et al. [22]	Hanske et al. [20]	Hemal et al. [34]	Klingler et al. [35]	Link et al. [36]	Lucas et al. [16]	Memon et al. [37]	Mohammed [38]	Olweny et al. [39]	Pahwa et al. [40]	Rivas et al. [15]	Simforoosh et al. [41]	Umari et al. [42]	Wang et al. [43]	Weise and Winfield [44]	Yanke et al. [21]
Success																										
Hypothesis/aim/objective clearly described	1	1	1	1	1	1	1	0	1	1	1	NA	1	1	NA	1	1	1	1	1	0	1	1	1	1	1
Main outcomes in Introduction or Methods	0	0	1	1	1	1	0	1	0	0	1	NA	0	1	NA	1	1	1	1	1	1	1	1	1	1	1
Patient characteristics clearly described	0	0	1	1	1	1	0	1	0	1	1	NA	1	1	NA	0	1	0	1	0	1	0	1	1	1	1
Interventions of interest clearly described	1	0	1	0	0	1	0	1	1	1	1	NA	1	1	NA	1	0	0	1	0	1	0	0	0	1	1
Principal confounders clearly described	0	0	1	0	0	0	0	0	0	0	0	NA	0	0	NA	1	1	0	0	0	1	0	1	0	1	2
Main findings clearly described	1	0	1	1	1	1	1	1	1	1	1	NA	0	0	NA	1	1	1	1	1	1	1	1	1	1	1
Estimates of random variability provided for main outcomes	0	0	1	0	1	1	1	1	0	0	1	NA	1	1	NA	1	0	1	1	0	1	0	0	1	0	1
All adverse events of intervention reported	1	0	1	1	0	1	0	1	1	1	0	NA	1	1	NA	0	1	0	0	1	1	1	1	0	1	0
Characteristics of patients lost to follow-up described	0	0	0	0	0	0	0	0	0	0	0	NA	0	0	NA	1	0	0	0	0	0	0	1	1	0	0
Probability values reported for main outcomes	1	1	1	1	0	0	0	0	0	0	0	NA	0	0	NA	1	0	0	0	0	0	0	0	1	0	1
Subjects asked to participate were representative of source population	0	0	0	0	0	1	0	0	1	1	0	NA	1	1	NA	1	1	0	1	1	1	0	1	0	1	1
Subjects prepared to participate were representative of source population	0	0	0	0	1	1	0	0	1	1	0	NA	1	1	NA	1	0	0	1	1	1	1	1	1	1	1
Location and delivery of study treatment was representative of source population	1	1	1	1	1	1	1	0	1	1	1	NA	1	1	NA	1	1	1	1	1	1	1	1	1	1	1
Study participants blinded to treatment	0	0	0	0	0	0	0	0	0	0	0	NA	0	0	NA	0	0	0	0	0	0	0	0	0	0	0
Blinded outcome assessment	0	0	0	0	0	0	0	0	0	0	0	NA	0	0	NA	0	0	0	0	0	0	0	0	0	0	0
Any data dredging clearly described	0	0	0	0	0	0	0	0	0	0	0	NA	0	0	NA	1	0	0	0	0	1	0	0	0	0	0
Analyses adjust for differing lengths of follow-up	0	0	0	0	0	0	0	0	0	0	0	NA	0	0	NA	1	0	1	0	0	0	0	0	0	0	1
Appropriate statistical tests performed	1	0	1	0	1	0	0	1	0	1	1	NA	0	0	NA	1	0	0	1	0	1	1	0	1	1	1
Compliance with interventions was reliable	0	0	0	0	0	0	0	0	0	0	0	NA	1	1	NA	1	1	1	1	1	1	1	1	1	1	1
Outcome measures were reliable and valid	1	0	1	0	1	0	0	0	0	1	1	NA	0	0	NA	1	1	0	1	0	1	0	1	1	1	1
All participants recruited from the same source population	1	1	1	1	0	1	1	0	1	1	1	NA	1	1	NA	1	1	1	1	1	1	0	1	1	1	1
All participants recruited over the same time period	1	1	1	1	0	0	0	0	1	0	1	NA	0	1	NA	0	1	1	0	0	0	1	1	1	0	1
Participants randomized to treatment(s)	0	0	0	0	0	0	0	0	0	0	0	NA	0	0	NA	0	0	1	0	0	0	0	0	0	0	0
Allocation of treatment concealed from investigators and participants	0	0	0	0	0	0	0	0	0	0	0	NA	0	0	NA	0	0	0	0	0	1	0	0	0	0	0
Adequate adjustment for confounding	0	0	0	0	0	0	0	0	0	0	0	NA	0	0	NA	0	0	0	0	0	0	0	0	0	0	1
Losses to follow-up taken into account	0	0	0	0	0	0	0	0	0	0	0	NA	0	0	NA	1	0	0	0	0	0	0	0	0	0	1
Study power	2	1	5	3	4	5	4	2	2	3	3	NA	4	3	NA	5	4	3	2	4	4	4	3	5	3	5
Sum	12	6	19	12	13	16	9	9	11	14	14	NA	14	15	NA	23	16	13	15	13	20	13	17	19	17	25
Complications																										
Hypothesis/aim/objective clearly described	1	1	1	1	1	1	1	0	1	1	1	1	1	1	1	1	1	NA	1	1	0	1	1	1	1	1
Main outcomes in Introduction or Methods	0	0	1	1	1	1	0	1	0	0	1	1	0	1	0	1	1	NA	1	1	1	1	1	1	1	1
Patient characteristics clearly described	0	0	1	1	1	1	0	1	0	1	1	1	1	1	1	0	1	NA	1	0	1	0	1	1	1	1
Interventions of interest clearly described	1	0	1	0	0	1	0	1	1	1	1	0	1	1	0	1	0	NA	1	0	1	0	0	0	1	1
Principal confounders clearly described	0	0	1	0	0	0	0	0	0	0	0	2	0	0	1	1	1	NA	0	0	1	0	1	0	1	2
Main findings clearly described	1	0	1	1	1	1	1	1	1	1	1	1	0	0	1	1	1	NA	1	1	1	1	1	1	1	1
Estimates of random variability provided for main outcomes	0	0	1	0	1	1	1	1	0	0	1	1	1	1	1	1	0	NA	1	0	1	0	0	1	0	1
All adverse events of intervention reported	1	0	1	1	0	1	0	1	1	1	0	1	1	1	0	0	1	NA	0	1	1	1	1	0	1	0
Characteristics of patients lost to follow-up described	0	0	0	0	0	0	0	0	0	0	0	0	0	0	0	1	0	NA	0	0	0	0	1	1	0	0
Probability values reported for main outcomes	1	1	1	1	0	0	0	0	0	0	0	1	0	0	0	1	0	NA	0	0	0	0	0	1	0	1
Subjects asked to participate were representative of source population	0	0	0	0	0	1	0	0	1	1	0	1	1	1	1	1	1	NA	1	1	1	0	1	0	1	1
Subjects prepared to participate were representative of source population	0	0	0	0	1	1	0	0	1	1	0	1	1	1	1	1	0	NA	1	1	1	1	1	1	1	1
Location and delivery of study treatment was representative of source population	1	1	1	1	1	1	1	0	1	1	1	1	1	1	0	1	1	NA	1	1	1	1	1	1	1	1
Study participants blinded to treatment	0	0	0	0	0	0	0	0	0	0	0	0	0	0	0	0	0	NA	0	0	0	0	0	0	0	0
Blinded outcome assessment	0	0	0	0	0	0	0	0	0	0	0	0	0	0	0	0	0	NA	0	0	0	0	0	0	0	0
Any data dredging clearly described	0	0	0	0	0	0	0	0	0	0	0	0	0	0	0	1	0	NA	0	0	1	0	0	0	0	0
Analyses adjust for differing lengths of follow-up	0	0	0	0	0	0	0	0	0	0	0	0	0	0	0	1	0	NA	0	0	0	0	0	0	0	1
Appropriate statistical tests performed	1	0	1	0	1	0	0	1	0	1	1	1	0	0	0	1	0	NA	1	0	1	1	0	1	1	1
Compliance with interventions was reliable	0	0	0	0	0	0	0	0	0	0	0	1	1	1	1	1	1	NA	1	1	1	1	1	1	1	1
Outcome measures were reliable and valid	1	0	1	0	1	0	0	0	0	1	1	1	0	0	1	1	1	NA	1	0	1	0	1	1	1	1
All participants recruited from the same source population	1	1	1	1	0	1	1	0	1	1	1	1	1	1	1	1	1	NA	1	1	1	0	1	1	1	1
All participants recruited over the same time period	1	1	1	1	0	0	0	0	1	0	1	1	0	1	1	0	1	NA	0	0	0	1	1	1	0	1
Participants randomized to treatment(s)	0	0	0	0	0	0	0	0	0	0	0	0	0	0	0	0	0	NA	0	0	0	0	0	0	0	0
Allocation of treatment concealed from investigators and participants	0	0	0	0	0	0	0	0	0	0	0	0	0	0	0	0	0	NA	0	0	1	0	0	0	0	0
Adequate adjustment for confounding	0	0	0	0	0	0	0	0	0	0	0	1	0	0	0	0	0	NA	0	0	0	0	0	0	0	1
Losses to follow-up taken into account	0	0	0	0	0	0	0	0	0	0	0	0	0	0	0	1	0	NA	0	0	0	0	0	0	0	1
Study power	2	1	5	3	4	5	4	2	2	3	3	5	4	3	1	5	4	NA	2	4	4	4	3	5	3	5
Sum	12	6	19	12	13	16	9	9	11	14	14	23	14	15	12	23	16	NA	15	13	20	13	17	19	17	25

*NA* not applicable

### Network meta-analyses for different outcomes

#### Network meta-analysis of operative success

**Fig. 3:**
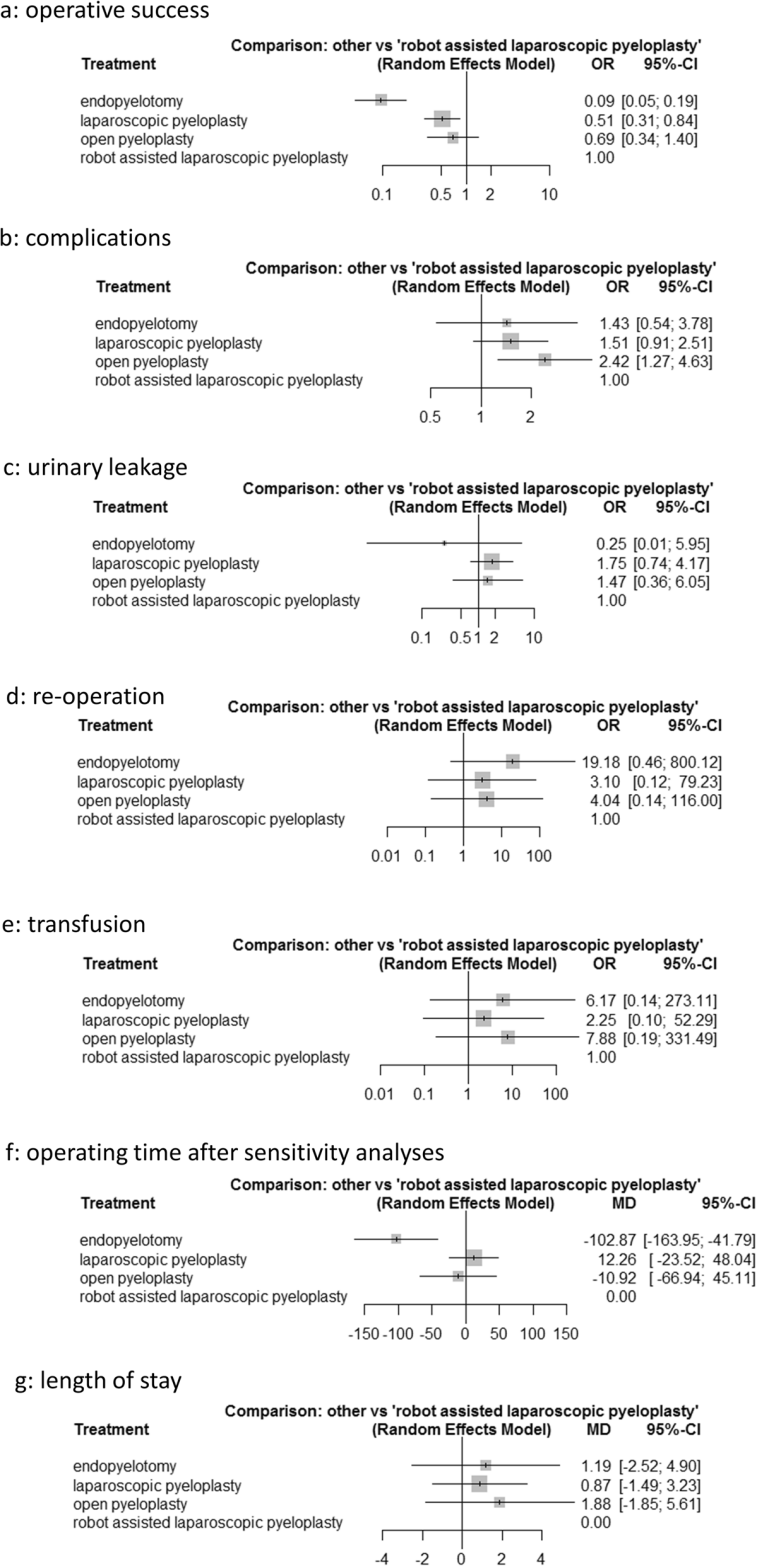
Pooled estimates for each endpoint. **a**) operative success. **b**) complications. **c**) urinary leakeage. **d**) re-operation. **e**) transfusion. **f**) operating time after sensitivity analyses. **g**) length of stay

The analysis of operative success included 34 pairwise comparisons from 24 studies. Compared with RP, EP and LP showed lower success rates with OR = 0.09 (95%CI 0.05–0.19; *p* < 0.001) for EP and OR = 0.51 (95%CI0.31–0.84; *p* = 0.008) for LP. No statistically significant difference was evident comparing OP and RP (OR = 0.69, 95%CI 0.34–1.4; *p* = 0.306). Table 3 depicts all pairwise comparisons in a league table. Associated p-scores are presented in Table 4. There was no evidence of study heterogeneity (I^2^ = 0%, *p* = 0.9041). Neither Cochran’s Q (Q = 1.98; *p* = 0.9213) nor the net heat plot depicted inconsistencies. Comparisons for OP, RP, LP, and EP as forest-plot are shown in Fig. 3a. Upon sensitivity analyses including only those studies with at least 12 months follow-up time (*n* = 13), comparable results were obtained (Additional file 1: Table S1). A total of 15 studies provided information on primary versus secondary UPJO [14, 16, 21, 22, 25, 27–29, 35–37, 39–41, 44]. Of these, only 9 studies explicitly included secondary UPJO [16, 21, 22, 25, 27, 28, 35, 37, 44]: Only 2 studies compared operative success between primary and secondary UPJO [25, 30]. Baldwin et al. stated, that for LP operative success was higher in the group with secondary UPJO (100% vs 89%). But for EP operative success was higher among patients with primary UPJO [25]. Calvert et al. reported higher success rates among patients with primary UPJO for LP (98% vs. 57%) and OP (96% vs 67%) [29].

**Table 3:** League table showing pairwise comparisons for all surgical approaches and end points included in the quantitative network meta-analysis

End point		Endopyelotomy (reference)	Laparoscopic pyeloplasty (reference)	Open pyeloplasty (reference)	Robot assisted laparoscopic pyeloplasty (reference)
operative success (OR)	EP (comparator)	1 (1–1), *p* = NA	**0.18 (0.11–0.31),** ***p*** **= < 0.001**	**0.14 (0.07–0.27),** ***p*** **= < 0.001**	**0.09 (0.05–0.19),** ***p*** **= < 0.001**
	LP (comparator)	**5.44 (3.26–9.08),** ***p*** **= < 0.001**	1 (1–1), *p* = NA	0.74 (0.44–1.26), *p* = 0.27	**0.51 (0.31–0.84),** ***p*** **= 0.008**
	OP (comparator)	**7.31 (3.72–14.39),** ***p*** **= < 0.001**	1.34 (0.8–2.27), *p* = 0.27	1 (1–1), *p* = NA	0.69 (0.34–1.4), *p* = 0.306
	RP (comparator)	**10.59 (5.25–21.37),** ***p*** **= < 0.001**	**1.94 (1.19–3.19),** ***p*** **= 0.008**	1.45 (0.71–2.94), *p* = 0.306	1 (1–1), *p* = NA
		EP (reference)	LP (reference)	OP (reference)	RP (reference)
overall complications (OR)	EP (comparator)	1 (1–1), *p* = NA	0.95 (0.41–2.18), *p* = 0.898	0.59 (0.25–1.39), *p* = 0.226	1.43 (0.54–3.78), *p* = 0.474
	LP (comparator)	1.06 (0.46–2.43), *p* = 0.898	1 (1–1), *p* = NA	**0.62 (0.41–0.95),** ***p*** **= 0.027**	1.51 (0.91–2.51), *p* = 0.115
	OP (comparator)	1.7 (0.72–4.01), *p* = 0.226	**1.61 (1.05–2.45),** ***p*** **= 0.027**	1 (1–1), *p* = NA	**2.42 (1.27–4.63),** ***p*** **= 0.007**
	RP (comparator)	0.7 (0.26–1.85), *p* = 0.474	0.66 (0.4–1.1), *p* = 0.115	**0.41 (0.22–0.79),** ***p*** **= 0.007**	1 (1–1), *p* = NA
		EP (reference)	LP (reference)	OP (reference)	RP (reference)
urinary leakage (OR)	EP (comparator)	1 (1–1), *p* = NA	0.14 (0.01–3.01), *p* = 0.211	0.17 (0.01–3.41), *p* = 0.247	0.25 (0.01–5.95), *p* = 0.392
	LP (comparator)	6.98 (0.33–146.48), *p* = 0.211	1 (1–1), *p* = NA	1.19 (0.39–3.62), *p* = 0.761	1.75 (0.74–4.17), *p* = 0.206
	OP (comparator)	5.87 (0.29–117.38), *p* = 0.247	0.84 (0.28–2.56), *p* = 0.761	1 (1–1), *p* = NA	1.47 (0.36–6.05), *p* = 0.591
	RP (comparator)	3.99 (0.17–94.46), *p* = 0.392	0.57 (0.24–1.36), *p* = 0.206	0.68 (0.17–2.79), *p* = 0.591	1 (1–1), *p* = NA
		EP (reference)	LP (reference)	OP (reference)	RP (reference)
re-operation (OR)	EP (comparator)	1 (1–1), *p* = NA	6.18 (0.97–39.29), *p* = 0.053	4.75 (0.7–32.39), *p* = 0.112	19.18 (0.46–800.12), *p* = 0.121
	LP (comparator)	0.16 (0.03–1.03), *p* = 0.053	1 (1–1), *p* = NA	0.77 (0.32–1.85), *p* = 0.557	3.1 (0.12–79.23), *p* = 0.494
	OP (comparator)	0.21 (0.03–1.43), *p* = 0.112	1.3 (0.54–3.14), *p* = 0.557	1 (1–1), *p* = NA	4.04 (0.14–116), *p* = 0.415
	RP (comparator)	0.05 (0–2.17), *p* = 0.121	0.32 (0.01–8.24), *p* = 0.494	0.25 (0.01–7.11), *p* = 0.415	1 (1–1), *p* = NA
		EP (reference)	LP (reference)	OP (reference)	RP (reference)
transfusion rate (OR)	EP (comparator)	1 (1–1), *p* = NA	2.74 (0.33–22.72), *p* = 0.35	0.78 (0.07–9.02), *p* = 0.844	6.17 (0.14–273.11), *p* = 0.347
	LP (comparator)	0.36 (0.04–3.03), *p* = 0.35	1 (1–1), *p* = NA	0.29 (0.04–2.16), *p* = 0.225	2.25 (0.1–52.29), *p* = 0.613
	OP (comparator)	1.28 (0.11–14.72), *p* = 0.844	3.5 (0.46–26.44), *p* = 0.225	1 (1–1), *p* = NA	7.88 (0.19–331.49), *p* = 0.279
	RP (comparator)	0.16 (0–7.18), *p* = 0.347	0.44 (0.02–10.32), *p* = 0.613	0.13 (0–5.34), *p* = 0.279	1 (1–1), *p* = NA
		EP (reference)	LP (reference)	OP (reference)	RP (reference)
operating time (mean minutes and SD) after sensitivity analyses	EP (comparator)	0 (0–0), *p* = NA	**−127.51 (−179.83--75.19),** ***p*** **= < 0.001**	**−76.16 (− 142.85--9.47),** ***p*** **= 0.025**	**−115.39 (− 175.19--55.58),** ***p*** **= < 0.001**
	LP (comparator)	**127.51 (75.19–179.83),** ***p*** **= < 0.001**	0 (0–0), *p* = NA	**51.35 (10–92.7),** ***p*** **= 0.015**	12.12 (− 16.84–41.08), *p* = 0.412
	OP (comparator)	**76.16 (9.47–142.85),** ***p*** **= 0.025**	**−51.35 (−92.7--10),** ***p*** **= 0.015**	0 (0–0), *p* = NA	−39.22 (−89.71–11.26), *p* = 0.128
	RP (comparator)	**115.39 (55.58–175.19),** ***p*** **= < 0.001**	−12.12 (−41.08–16.84), *p* = 0.412	39.22 (− 11.26–89.71), *p* = 0.128	0 (0–0), *p* = NA
		EP (reference)	LP (reference)	OP (reference)	RP (reference)
length of stay (mean days and SD)	EP (comparator)	0 (0–0), *p* = NA	0.32 (−2.55–3.18), *p* = 0.827	−0.69 (−4.76–3.37), *p* = 0.739	1.19 (− 2.52–4.9), *p* = 0.53
	LP (comparator)	−0.32 (−3.18–2.55), *p* = 0.827	0 (0–0), *p* = NA	−1.01 (− 3.89–1.87), *p* = 0.492	0.87 (− 1.49–3.23), *p* = 0.471
	OP (comparator)	0.69 (− 3.37–4.76), *p* = 0.739	1.01 (− 1.87–3.89), *p* = 0.492	0 (0–0), *p* = NA	1.88 (− 1.85–5.61), *p* = 0.323
	RP (comparator)	−1.19 (− 4.9–2.52), *p* = 0.53	−0.87 (− 3.23–1.49), *p* = 0.471	−1.88 (− 5.61–1.85), *p* = 0.323	0 (0–0), *p* = NA

Estimates with corresponding statistically significant p-values (*p* < 0.05) are written in boldface

**Table 4:** P-scores ranking the surgical approaches for every outcome based on the amount of certainty that a single treatment outperforms the average of competing interventions. The p-score ranges from 0 to 1, the latter indicating the highest certainty possible

	Endopyelotomy	Laparoscopic pyeloplasty	Open pyeloplasty	Robot assisted laparoscopic pyeloplasty
Operative success	0.0000	0.3797	0.6727	0.9476
Few overall complications	0.5583	0.4975	0.0435	0.9007
Few cases of urinary leakage	0.8584	0.1963	0.3462	0.5991
Low re-operation rate	0.0476	0.6472	0.4768	0.8284
Low transfusion rate	0.3089	0.6730	0.2247	0.7934
Short operating time after sensitivity analyses	0.9958	0.0712	0.6471	0.2860
Short length of stay	0.4365	0.5252	0.2589	0.7794

#### Network meta-analysis of overall complications

The network meta-analysis of overall complications included 31 pairwise comparisons from 23 studies. Compared with OP, LP (OR = 0.62; 95%CI 0.41–0.95; *p* = 0.027) as well as RP (OR = 0.41; 95%CI 0.22–0.79; *p* = 0.007) had a statistically lower risk for complications. No statistically significant difference was detected comparing EP and OP. All pairwise comparisons are depicted in Table 3. Associated p-scores are presented in Table 4. The study heterogeneity was potentially irrelevant (I^2^ = 13.7%, *p* = 0.1416). Neither Cochran’s Q (Q = 1.02; *p* = 0.9064) nor the net heat plot depicted inconsistencies. Figure 3b depicts comparisons for OP, RP, LP, and EP.

#### Network meta-analysis of urinary leakage

The network meta-analysis of urinary leakage included 9 pairwise comparisons from 7 different studies. Compared with RP none of the other surgical treatment options had a statistically significant higher or lower risk for urinary leakage. Table 3 summarizes these findings, and Table 4 depicts the associated p-scores. There was no evidence of study heterogeneity (I^2^ = 0%, *p* = 0.5161). No inconsistencies were depicted by Cochran’s Q (Q = 0,58; *p* = 0.4471) or the net heat plot. Comparisons for OP, RP, LP, and EP as forest-plot are shown in Fig. 3c.

#### Network meta-analysis of re-operation

The analysis of re-operation was based on 10 pairwise comparisons from 8 studies. Compared with RP, none of the other surgical treatment options had a statistically significant higher or lower risk for re-operation. All pairwise comparisons are depicted in Table 3. Associated p-scores are presented in Table 4. There was no evidence of study heterogeneity (I^2^ = 0%, *p* = 0.3001). Neither Cochran’s Q (Q = 0.02; *p* = 0.9897) nor the net heat plot revealed inconsistencies. Figure 3d depicts comparisons for OP, RP, LP, and EP. Upon sensitivity analyses including only those studies with at least 12 months follow-up time (*n* = 13), comparable results were obtained and are depicted in Additional file 1: Table S2 [8, 14, 15, 21, 22, 28, 30, 33–35, 38, 40, 42]. Five studies stated, whether concomitant stones were present at the time of pyeloplasty but only 2 studies evaluated any effect on one of the outcomes [15, 16, 27, 28, 42]. Lucas et al. reported that presence of urolithiasis did not affect the rate of secondary interventions [16].

#### Network meta-analysis of transfusion rate

The analysis of transfusion rates included 11 pairwise comparisons from 7 studies. Compared with RP none of the other surgical treatment strategies reached a statistically significant difference in transfusion rates as presented in Table 3. Associated p-scores are depicted by Table 4. The study heterogeneity was “moderate” but not statistically significant (I^2^ = 42.3.7%, *p* = 0.4396). There were statistically significant inconsistencies (Cochran’s Q = 6.64, *p* = 0.0361) for which the sources were not identifiable by visual assessment of the net heat plot. Therefore, no sensitivity analyses excluding any studies were possible. Comparisons for OP, RP, LP and EP are depicted by Fig. 3e.

#### Network meta-analysis of operating time

A total of 14 pairwise comparisons from 12 studies were included in the analysis of operating time. Compared with EP, RP had a statistically significant longer operating time: mean = 102.87 min (95%CI 41.79 min–163.95 min, *p* = < 0.001). Further statistically significant differences in operating times were detected when comparing LP to EP with mean = 115.13 min (95%CI 65.63 min–164.63 min, *p* = < 0.001) and OP to EP with mean = 91.96 min (95%CI 32.33 min–151.58 min, *p* = 0.003). No statistically significant differences resulted from comparisons of the operative techniques against each other. The study heterogeneity was “considerable” and statistically significant (I^2^ = 95.2%, *p* < 0.001). The net heat plot suggested that the study design comparing EP, LP, and OP contributed most to network inconsistencies (Q = 101.59, *p* < 0.001). In consequence, sensitivity analyses excluding the study of Chen et al. were conducted. This resulted in 11 pairwise comparisons from 11 studies. Again, RP had a statistically significant longer operating time when compared to EP mean = 115.39 min (95%CI 55.58 min–175.19 min, *p* = < 0.001). This was also true for comparisons of LP or OP to EP. For LP to EP with mean = 127.51 min (95%CI 75.19 min - 179.83 min, *p* = < 0.001) and for OP to EP mean 76.16 min (95%CI 9.47 min–142.85 min, *p* = 0.025). In addition, statistically significant longer operating times resulted from a comparison of LP to OP with mean = 51.35 min (95%CI 10 min–92.7 min, *p* = 0.015). Table 3 depicts all pairwise comparisons of the reduced analysis. Associated p-scores of the reduced model are detailed in Table 4. The aforementioned “considerable” heterogeneity remained (I^2 = 92.4%, *p* < 0.001) which was not explored further in order to maintain adequate numbers of comparisons. As only direct comparisons remained in the reduced analysis, no evaluation of inconsistencies was reasonable. Bird et al. reported that concurrent treatment of nephrolithiasis did not affect operating time [27]. Figure 3f depicts comparisons for OP, RP, LP, and EP after sensitivity analyses.

#### Network meta-analysis of length of stay

The analysis of length of stay included 8 pairwise comparisons from 8 studies. Compared with RP none of the surgical treatment options had a statistically significant shorter or longer length of stay. All pairwise comparisons are depicted in Table 3 whereas Table 4 shows the associated p-scores. Analyses of heterogeneity revealed an I^2^ of 96.7% (*p* < 0.001). Due to the available study designs with direct comparisons only, no evaluation of inconsistency was warranted. Again, no further subgroup analyses were performed because of low numbers of comparisons. Comparisons for OP, RP, LP, and EP as forest-plot are shown in Fig. 3g.

### Publication bias

Assessment of publication bias was possible for operative success and complications with the interventions LP versus OP and LP versus RP each. On visual assessment, slight asymmetry was evident for operative success with the comparison of LP versus OP. Less studies reported on ORs < 0.74. Statistical evaluation did not reveal significant publication bias (*p* = 0.9118). Visual assessment of publication bias for operative success with RP compared to LP showed slight publication bias as well. Less studies reported on ORs > 1.94. Again, statistical evaluation did not reveal significant publication bias (*p* = 0.1519). Moreover, visual assessment comparing LP versus OP with respect to complications also resulted in the impression of slight publication bias which was not statistically significant: *p* = 0.365. Fewer studies reported on ORs < 0.62.Visual assessment and statistical evaluation of the comparison of LP and RP yielded no publication bias (*p* = 0.4808). Figure S1 in the Additional file 1 illustrates the funnel plots.

### Narrative meta-analysis of other outcomes

Only three studies reported on suturing time for RP versus LP [27, 33, 34]. In all cases, suturing time was shorter for RP. Estimated blood loss was reported by 13 studies of which only 4 studies provided estimates of dispersion which did not allow for meta-analyses. In most studies, EP had the lowest blood loss, followed by LP and RP. The highest blood loss was reported for OP in all studies. Due to the nature of the intervention, conversion rates were only reported for RP and LP: Low event rates did not allow for any reliable pooling which applied to death rates as well. Analgesia requirement was reported by 7 studies with different medication such as morphine equivalents, diclophenac, pethidine or tramadol and most of the time without measure of dispersion. Overall, the studies reported EP to have the lowest analgesic requirement, followed by LP and OP. Only one study compared RP and LP and described lower need for analgesic medication for RP. For time to return to normal activity, renal function, and costs only 1 to 3 studies reported estimates with heterogeneous outcome definitions. Therefore, no meta-analysis was possible in these cases.

## Discussion

Several surgical techniques have been developed for treatment of UPJO, each yielding unique advantages and potential limitations. Although RP and LP are allegedly superior, these claims are based on pairwise meta-analyses that failed to evaluate all available techniques at once. In contrast, our study provides a comprehensive overview on OP, EP, LP and RP, comparing their performance with respect to crucial clinical outcomes. Our results indicate that RP is the technique with highest rates of operative success, lowest overall complication rates, shortest hospital stay as well as lowest re-operation rates and transfusion rates. On the other hand, EP yields lowest rates of urinary leakage and shortest operating times. Robot assisted surgery is known for its minimally invasive nature which goes along with less postoperative pain and earlier recovery, which probably causes shorter hospital stays. In addition, robot assisted surgery allows for high precision movements with articulated arms and provides magnified 3-D vision for the surgeon [45]. This might explain high operative success rates of RP. Low transfusion rates of RP are the consequence of the minimally invasive nature of robotic surgery which allows immediate and precise reaction to local bleeding [46]. The reason for short operating time in EP probably is that the kidney access requires more time during abdominal surgery than this endoscopic procedure. In addition, EP is less complex, even though the percutaneous approach involves a short flank incision and sewing techniques which are both of low complexity [47, 48]. Another advantage of EP are low rates of urinary leakage, which might be due to the small extent of manipulation compared to pyeloplasty approaches. However, the reduced invasiveness of EP is on the cost of high recidive rates. Urothelial scarring probably explains these differences since EO only involves an urothelial incision as opposed to surgical approaches where strictured tissue is resected [49, 50]. When evaluating different treatment approaches, costs have to be taken into account as well. Only one of the included studies evaluated treatment costs [36]: Link et al. reported 2.7 times higher costs for RP ($5323.80) compared to LP ($1989.87). More literature is available comparing LP, RP and OP: Yu et al. found RP to be associated with the highest median costs ($11,829), followed by OP ($9520) and LP ($8291) [51]. Gettmann et al. published costs for EP ranging between $3842 and $5297 compared to higher costs for LP ($7026) and OP ($7119) [52]. No decision tree analyses have been published to evaluate whether the differences in these expenditures outbalance the benefits of the approaches. Overall study quality was moderate due to limitations in study design such as randomization, concealment of treatment allocation, blinding and omission of multivariable analyses. Still, the nature of interventions and outcomes assessed in this meta-analysis questions, whether higher quality trials would yield relevant changes in the observed effects. Our study is not devoid of limitations, which are mainly inherent to the published trials as its data source: most publications did not adjust for confounding and only one randomized controlled trial could be included. Therefore, the pooled estimates might slightly vary from the true effects. Visual assessment of publication bias yielded minor asymmetries for some the funnel plots. Nevertheless, statistical tests did not return statistically significant evidence for publication bias, which does not completely exclude such bias but suggests low impact. The network meta-analyses of operating time and length of stay yielded statistically significant heterogeneity which could not be bypassed by subgroup analyses in order to maintain adequate numbers of comparisons. Therefore, the pooled estimates might not be generalizable to specific patient subpopulations. Finally, results on the inferiority of EP might be due to differing failure patterns, which is mainly due to missed diagnosis of crossing vessels in EP, and due to inadequate spatulation or incomplete excision of the diseased segment in RP, LP and OP. Still, EP studies were included since they contributed indirect evidence for comparison of other surgical approaches as well. Still, our findings are based on a total of 26 included studies which is the largest meta-analysis published so far and the first comparing more than two interventions simultaneously. The novel network meta-analyses approach further allows for combination of direct and indirect evidence to enhance comparisons of formerly underpowered treatment approaches [53–55].

## Conclusions

Comparing OP, EP, LP and RP for UPJO in a comprehensive network meta-analysis approach, our study found that RP has the highest rates of operative success and as well as LP lower complication rates than OP. Operating time is shortest for EP, followed by OP, RP, and LP. Surgeons should consider these findings when selecting the optimal treatment method for individual UPJO patients. Further research should aim for improvement of study quality and decision tree analyses based on associated costs.

## Supplementary information

**Additional file 1: Figure S1.** Funnel plots for operative success and complications with different comparisons **Table S1.** Operative success based on studies with at least 12 months follow-up (*n* = 13) **Table S2.** Probability of re-operation based on studies with at least 12 months follow-up (*n* = 13). **Table S3.** Study quality according to the Newcastle Ottawa Scale.

## Data Availability

The extracted study’data and statistical code used for the current study are available from the corresponding author on reasonable request.

## Abbreviations

EP: Endopyelotomy
IVU: Intravenous urography
LP: Laparoscopic pyeloplasty
OP: Open pyeloplasty
RP: Robotic assisted pyeloplasty
UPJO: Ureteropelvic junction obstruction
