# Supplementary material for: Surgical approaches for treatment of ureteropelvic junction obstruction – a systematic review and network meta-analysis
Source: BMC Urol. 2019 Nov 11;19:112. doi: 10.1186/s12894-019-0544-7 (PMC6849262; doi:10.1186/s12894-019-0544-7)
Supplement: Supplementary file 1 — Additional file 1: Figure S1. Funnel plots for operative success and complications with different comparisons Table S1. Operative success based on studies with at least 12 months follow-up (n = 13) Table S2. Probability of re-operation based on studies with at least 12 months follow-up (n = 13). Table S3. Study quality according to the Newcastle Ottawa Scale. [file 12894_2019_544_MOESM1_ESM.docx]

**Supplemental material**

Figure S1: Funnel plots for operative success and complications with different comparisons


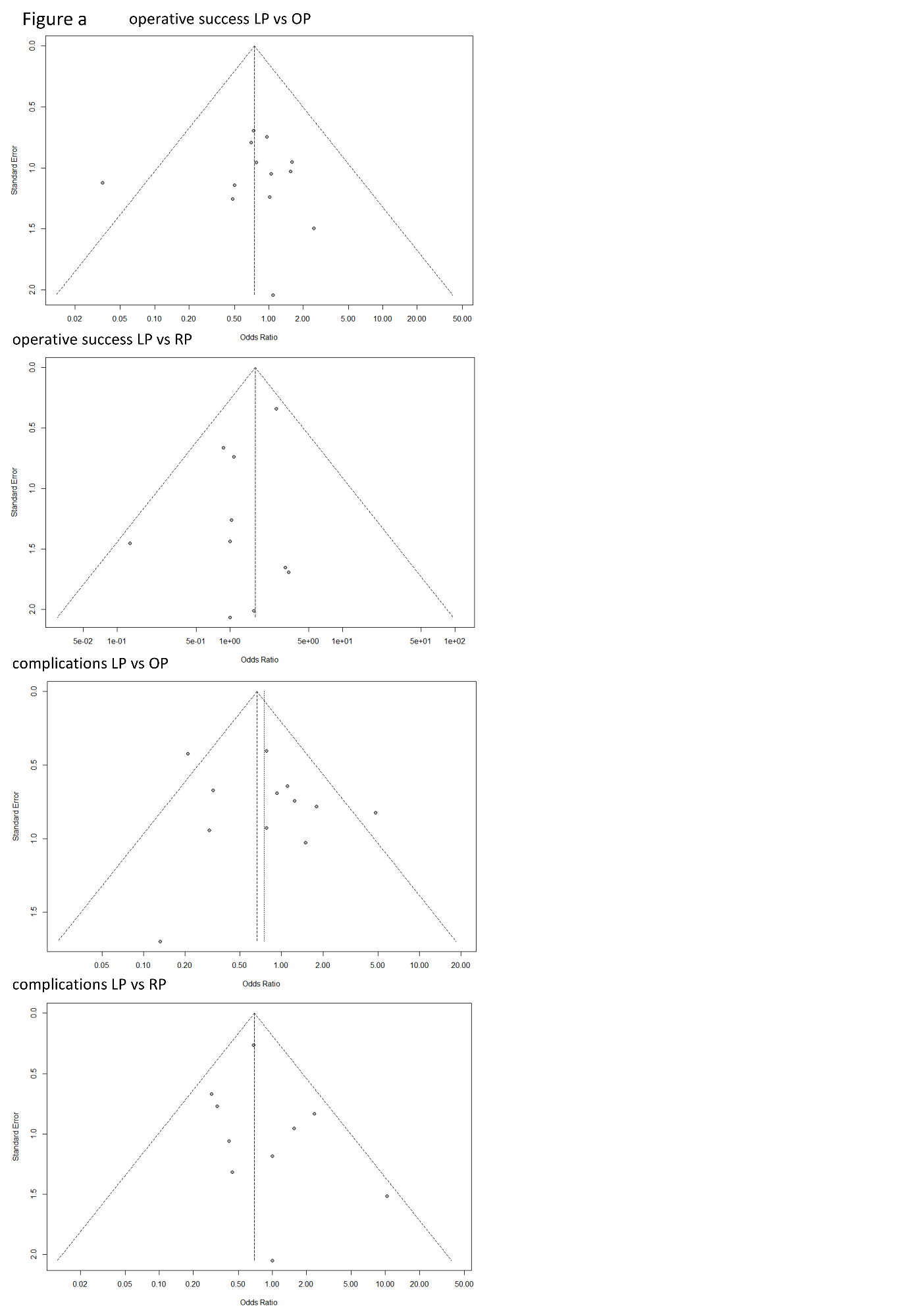


Table S1: Operative success based on studies with at least 12months follow-up (n=13).

|  | **endopyelotomy** | **laparoscopic pyeloplasty** | **open pyeloplasty** | **robot assisted laparoscopic pyeloplasty** |
| --- | --- | --- | --- | --- |
| endopyelotomy | 1 (1-1), p=NA | **0.18 (0.11-0.31), p=<0.001** | **0.16 (0.07-0.36), p=<0.001** | **0.08 (0.02-0.33), p=<0.001** |
| laparoscopic pyeloplasty | **5.44 (3.2-9.27), p=<0.001** | 1 (1-1), p=NA | 0.87 (0.44-1.75), p=0.704 | 0.44 (0.12-1.67), p=0.226 |
| open pyeloplasty | **6.23 (2.79-13.93), p=<0.001** | 1.14 (0.57-2.3), p=0.704 | 1 (1-1), p=NA | 0.5 (0.12-2.09), p=0.344 |
| robot assisted laparoscopic pyeloplasty | **12.41 (3.07-50.1), p=<0.001** | 2.28 (0.6-8.66), p=0.226 | 1.99 (0.48-8.3), p=0.344 | 1 (1-1), p=NA |

Table S2: Probability of re-operation based on studies with at least 12months follow-up (n=13).

|  | **endopyelotomy** | **laparoscopic pyeloplasty** | **open pyeloplasty** | **robot assisted laparoscopic pyeloplasty** |
| --- | --- | --- | --- | --- |
| endopyelotomy | 1 (1-1), p=NA | 6.12 (0.93-40.16), p=0.059 | 4.85 (0.63-37.59), p=0.13 | 18.98 (0.45-804.63), p=0.124 |
| laparoscopic pyeloplasty | 0.16 (0.02-1.07), p=0.059 | 1 (1-1), p=NA | 0.79 (0.2-3.15), p=0.742 | 3.1 (0.12-79.23), p=0.494 |
| open pyeloplasty | 0.21 (0.03-1.6), p=0.13 | 1.26 (0.32-5), p=0.742 | 1 (1-1), p=NA | 3.91 (0.12-132.21), p=0.448 |
| robot assisted laparoscopic pyeloplasty | 0.05 (0-2.23), p=0.124 | 0.32 (0.01-8.24), p=0.494 | 0.26 (0.01-8.65), p=0.448 | 1 (1-1), p=NA |

Table S3: study quality according to the Newcastle Ottawa Scale

|  | Baldwin et al (25) | Bernie et al (26) | Bird et al (27) | Brooks et al (28) | Calvert et al (29) | Chen et al (30) | Danuser et al (31) | Desai et al (14) | Fahad (32) | Garcia-Galisteo et al (33) | Han et al (22) | Hanske et al (20) | Hemal et al (34) | Klingler et al (35) | Link et al (36) | Lucas et al (16) | Memon et al (37) | Mohammed et al (38) | Olweny et al (39) | Pahwa et al (40) | Rivas et al (15) | Simforoosh et al (41) | Umari et al (42) | Wang et al (8) | Weise and Winfield (44) | Yanke et al (21) |
| --- | --- | --- | --- | --- | --- | --- | --- | --- | --- | --- | --- | --- | --- | --- | --- | --- | --- | --- | --- | --- | --- | --- | --- | --- | --- | --- |
| Outcome: operative success | 4 | 4 | 4 | 4 | 4 | 5 | 4 | 3 | 5 | 5 | 4 | NA | 5 | 5 | NA | 6 | 4 | 4 | 5 | 5 | 5 | 4 | 5 | 5 | 5 | 7 |
| Outcome: complications | 4 | 4 | 4 | 4 | 4 | 5 | 4 | 3 | 5 | 5 | 4 | 6 | 5 | 5 | 5 | 6 | 4 | NA | 5 | 5 | 5 | 4 | 5 | 5 | 5 | 7 |

**search strategy:**

**Medline and Embase via OVID:**

(exp pyeloplasty/ or pyeloplasty.ab,ti.)

and

(exp laparoscopy/ or exp robot assisted surgery/ or exp robot surgery/ or exp robotics/ or exp robotic surgical procedure/ or exp open surgery/ or laparoscopy.ab,ti. or robot assisted surgery.ab,ti. or robot surgery.ab,ti. or robotics.ab,ti. or robotic surgical procedure.ab,ti. or open surgery.ab,ti.)

**Cochrane:**

#1 pyeloplasty

#2 laparoscopy

#3 robot assisted surgery

#4 robotics

#5 robot surgery

#6 robotic surgical procedure

#7 open surgery

#8 #2 or #3 or #4 or #5 or #6 or #7

#9 #8 and #1
